# Patient Survey of current water Intake practices in autosomal dominant Polycystic kidney disease: the SIPs survey

**DOI:** 10.1093/ckj/sfw153

**Published:** 2017-02-17

**Authors:** Ragada El-Damanawi, Tess Harris, Richard N. Sandford, Fiona E. Karet Frankl, Thomas F. Hiemstra

**Affiliations:** 1 Cambridge Clinical Trials Unit, Cambridge Biomedical Campus, Cambridge UK; 2 PKD charity, London, UK; 3 Department of Medical Genetics, Cambridge Institute for Medical Research, Addenbrookes Hospital, Hills Road, Cambridge, UK; 4 Division of Renal Medicine and Division of Experimental Medicine, University of Cambridge, Cambridge, UK; 5 Division of Experimental Medicine, University of Cambridge, Cambridge, UK

**Keywords:** autosomal dominant, clinical trials, polycystic kidney disease, water, vasopressin

## Abstract

**Background:** Autosomal dominant polycystic kidney disease (ADPKD) affects 12.5 million worldwide. Vasopressin drives cysts growth and in animal models can be suppressed through high water intake. A randomized controlled trial of ‘high’ versus ‘standard’ water intake in ADPKD is essential to determine if this intervention is beneficial. We conducted an ADPKD patient survey to gain an understanding of current fluid intake practices and the design challenges of a randomized water intake trial.

**Methods:** In collaboration with the PKD Charity, we developed and distributed an online survey to ADPKD patients over age 16 years and not on renal replacement therapy.

**Results:** Of the 2377 invited, 89 ADPKD patients completed the Survey of current water Intake practices in autosomal dominant Polycystic kidney disease (SIPs) online questionnaire. Most were female (65, 73%) and white (84, 94%), with a median age group of 45–49 years. The risk of contamination between treatment arms was highlighted by the survey as the majority (70, 79%) routinely discussed ADPKD management with family despite only 17% sharing the same household. More participants reported drinking beyond thirst (65, 73%) than those actually indicating a daily fluid intake of >2 L (54, 61%). This discrepancy emphasizes inaccuracies of fluid intake estimates and the requirement for objective methods of measuring water intake. Overall, only 51% believed high water intake was beneficial, while 91% were willing to participate in research evaluating this.

**Conclusion:** ADPKD poses unique design challenges to a randomized water intake trial. However, the trial is likely to be supported by the ADPKD community and could impact significantly on PKD management and associated healthcare costs.

## Introduction

Autosomal dominant polycystic kidney disease (ADPKD) is the most common inherited renal disease, affecting 12.5 million people worldwide [1]. It accounts for 7% of incident adults commencing renal replacement therapy in the UK [2]. ADPKD is characterized by the relentless growth of cysts throughout the kidneys, resulting in progressive kidney dysfunction over time. Half of those affected require dialysis by 50 years of age. In the early stages of the disease, there is compensatory hyper-filtration by the remaining normal nephrons, preserving renal excretory function. Over time, these mechanisms are overwhelmed due to ongoing cyst growth causing compression, fibrosis and inflammation. This results in progressive renal impairment [3].

Most cases are caused by mutations in the *PKD1* or *PKD2* genes, but in up to 15% of cases no genetic mutation is identified using currently available sequencing technology [1]. Although an autosomal dominant condition, the initial germ-line mutation is accompanied by further somatic mutations that cause a ‘second hit’ required for cystogenesis [4], together with interactions with modifier genes and environmental factors. These are primarily responsible for phenotypic variability even within families.

The pathogenic role of vasopressin in ADPKD has been shown in previous studies [5, 6], and vasopressin receptor antagonists have emerged as a novel treatment that slows PKD progression. In the TEMPO3:4 trial, the V2 receptor antagonist tolvaptan resulted in reduced growth in total kidney volume (TKV) compared with placebo (2.8% versus 5.5% per year), and a lower rate of worsening kidney function [7]. However, 23% discontinued the drug due to side effects including aquaresis and liver toxicity. Given that vasopressin release can be readily suppressed by drinking beyond thirst [6, 8–10], it is possible that disease progression may similarly be slowed by high water consumption.

Animal studies of high water intake have been encouraging [11]. In contrast, human data are limited and conflicting, with one study showing potential harm [12]. Nonetheless, on this assumption, many clinicians encourage ADPKD patients to increase their daily water intake to 2–4 L. However, there is no existing evidence to support the population-wide promotion of high water intake in ADPKD. Against this background of uncertainty over the optimum fluid consumption, Tong *et al.* reported survey data showing that patients with ADPKD identify non-pharmacological interventions such as fluid intake and diet as key research priorities [13].

There is an urgent need for trials to evaluate the optimal hydration strategy in patients with ADPKD. We conducted a survey of patients with ADPKD to inform the design of a randomized feasibility trial of high versus standard water intake. In this study, we considered potential barriers to, and methodological issues involved in, conducting a water intake trial in patients with PKD.

## Materials and methods

We developed the Survey of current water Intake practices in autosomal dominant Polycystic kidney disease (SIPs survey) with the PKD Charity and patients with ADPKD. Draft questionnaires were screened for suitability and clarity by a patient panel, and amended as required before administration of the final survey. The final version (see Supplementary data) was distributed online via the PKD Charity website and on social media, and included questions on demographics, family relationships with particular reference to other affected family members, the extent to which patients discussed their condition and treatments with relatives, existing fluid consumption patterns, willingness to participate in a hypothetical randomized water intake trial and willingness to perform urine testing at home.

Participants were asked to report their estimated daily fluid intake. Based on these estimates, participants were divided into those with an intake >2 or ≤2 L/day. Responses were compared between the High Intake and Low Intake groups.

Data were reported as mean ± standard deviation or median (interquartile range, IQR) as appropriate for their distribution, unless otherwise specified. Categorical variables were compared using Fisher’s exact test. All data were summarized and analysed using Stata version 13 (Stata Corp., College Station, TX, USA).

## Results

We carried out a consultation process including clinicians, healthcare professionals, patients and methodologists in collaboration with the PKD Charity (http://pkdcharity.org.uk). We identified several areas that could be fully or partly addressed by directly surveying patients with ADPKD, including (i) willingness to participate in clinical research, (ii) willingness to carry out self-monitoring of urine parameters, (iii) existing water consumption practices and willingness to deviate from this in a randomized design and (iv) interaction and co-habitation with affected relatives (potential for contamination of the control arm). All ADPKD patients on the PKD Charity mailing list aged 16 years or older were eligible to participate in the SIPs survey.

### Participant characteristics

We recorded responses from 89 people with ADPKD. Of these, 65 (73%) were female and 84 (94%) were Caucasian. The median age group of participants was 45–49 years, with a median age group of 30–34 years at diagnosis.

The majority of respondents (52, 58%) were employed; a further 7 (8%) were students and 18 (20%) were retired; and 41 (46%) respondents completed the questionnaire online using a desktop or laptop computer, while 26 (29%) used a smartphone and 22 (25%) used a tablet computer.

**Fig. 1:**
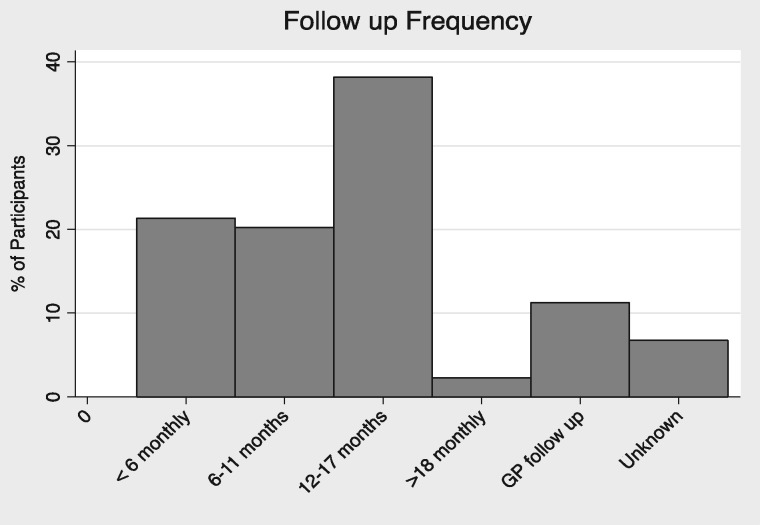
Follow-up site and frequency of respondents. The majority of respondents received follow-up at least every 18 months, 21% had follow-up more frequently than every 6 months. Only 11% received follow-up in primary care.

Seventy-three (82%) participants reported as receiving their medical care in secondary rather than primary care. The median follow-up frequency was 12–17 months (Figure 1), while only 11% (10/89) received their follow-up in primary care. The remaining 7% did not provide a response to the question.

#### Family relations

**Fig. 2:**
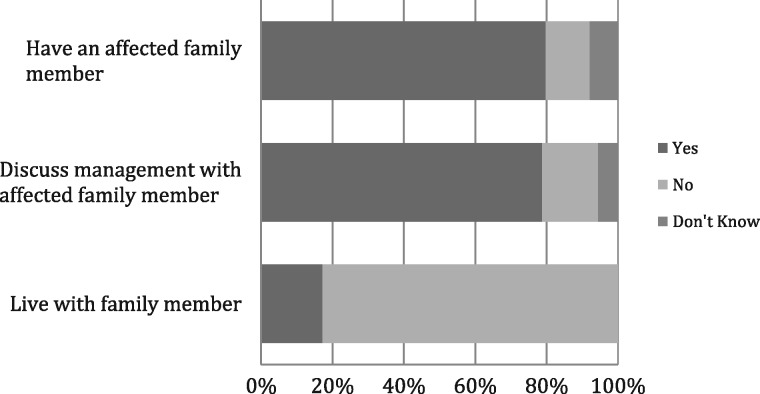
Responses to family relationship survey questions. The majority (80%) knew an affected family member and discussed the management of the condition with them (79%), despite most (83%) living in different households.

Seventy-one (80%) participants indicated that they had affected family members. Of the remainder, 11 (12%) reported no affected relatives and the remaining 7 (8%) did not know. Importantly, the majority (70, 79%) reported discussing their condition and treatment with affected family, despite only 15 participants (17%) sharing a home with them (Figure 2).

#### Current intake practices

**Fig. 3:**
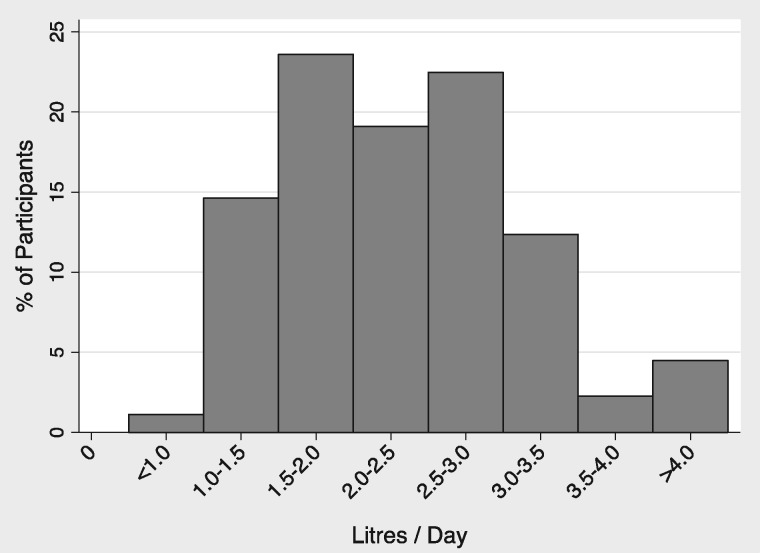
Estimated current daily fluid intake (L/day).There was a wide variation in daily fluid intake; however, the majority (61%) reported an intake of  ≥2 L/day with only 4% drinking > 4 L/day. The rest (39%) drank < 2 L/day.

There was wide variation in self-reported fluid intake among participants (ranging <1.0 to >4.0 L/day): 54 participants (61%) reported drinking >2 L/day (Figure 3). However, 65 patients (74%) reported drinking beyond thirst, yet 18 (28%) of these estimated their fluid intake at <2.0 L daily.

We next compared responses between patients reporting intake >2 L/day (the High Intake group) and those reporting ≤2 L/day (Low Intake group) as shown in Table 1. Significantly, more patients in the High Intake group (47/54, 87%) reported making an effort to drink beyond thirst compared with the Low Intake group (18/35, 51%, P = 0.001). The proportion of participants reporting nocturia did not differ between the High Intake and Low Intake groups [31/54 (57%) versus 22/35 (63%), P = 0.66]. A similar proportion of participants in the High Intake and Low Intake groups declared a belief that high water intake was beneficial [28/54 (52%) versus 17/35 (49%), P = 0.1].

**Table 1:** Comparison of water intake practices in High and Low Intake groups

Question	Low Intake	High Intake	P-value
	(≤2 L/day) (%)	(>2 L/day) (%)	
Do you actively make an effort to drink throughout the day even when you are not thirsty? (Yes)	51	87	0.001
	(18/35)	(47/54)	
Do you get up at night to pass urine? (Always/Frequent)	63	57	0.660
	(22/35)	(31/54)	
To what extent do you believe drinking water throughout the day can slow down the progression of PKD? (Agree)	49	52	0.100
	(17/35)	(28/54)	

### Research participation and self-monitoring

**Fig. 4:**
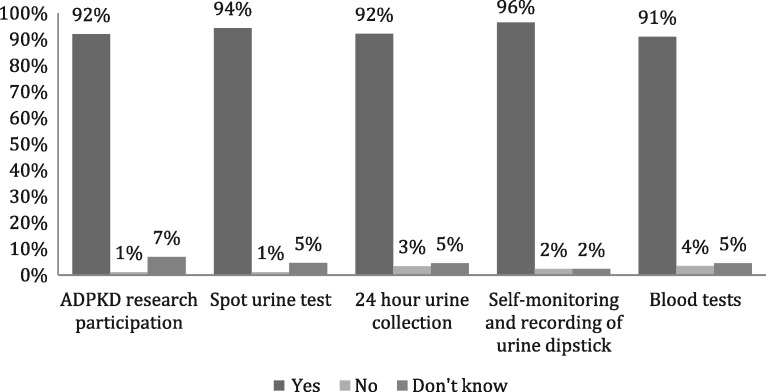
Respondents willingness to participate in various aspects of research. A total of 92% indicated they were willing to participate in research, while the majority agreed to different methods of urine testing and blood tests for the purposes of research; 96% were happy to perform self-monitoring and recording of urine results.

A very high proportion of respondents (80, 92%) indicated a willingness to participate in ADPKD research. Most respondents indicated that, as part of research, they would undertake 24-h (92%), spot (94%) or home urine testing (96%, Figure 4).

## Discussion

The results of the SIPs survey showed that in this group of ADPKD patients, respondents were highly motivated to participate in a water intake trial. They were also willing to collect 24-h urine samples, carry out home urine monitoring and provide blood samples. More than 9 in every 10 respondents expressed a willingness to undergo a range of urine assessments, and this willingness was not affected by the method of collection including 24-h urine collection. The majority (82%) received follow-up in secondary care.

The survey demonstrated a wide range of daily fluid intake volumes, suggesting that many PKD patients do not currently drink beyond thirst despite widely prevalent guidance from healthcare professionals and via other media to drink large volumes. We set a threshold for defining ‘high water intake’ of 2 L/day based on previous data showing a urine osmolality of 344–648 mOsm/kg in patients with PKD [8]. Given that the free water clearance formula allows the estimation of the requisite fluid intake to achieve a set urine osmolality, it follows that a patient with a urine osmolality of 344 mOsm estimated from a collection of 1.2 L of urine (assuming insensible losses of 500 mL) would have a requisite fluid intake of 2028 L to achieve a urine osmolality of 270 mOsm/kg.

$$Fluid\;intake=\frac{\left(uOsm)(uVol\right)}{Target\;uOsm}+Insensible\;Loss$$

It is therefore reasonable to accept 2 L as the lower limit of the ‘high water intake’ range.

Consistent with the wide distribution of fluid intake, only half of respondents held the belief that high water intake was beneficial, indicating equipoise at patient level. The discrepancy between the proportion of patients ‘drinking beyond thirst’ (74%) and those reporting intake >2 L/day (61%) suggests that patient estimates of fluid consumption are inaccurate. Objective estimates of fluid intake are therefore important for future studies.

Given the autosomal dominant inheritance of PKD, it is not surprising that the majority of survey respondents had an affected family member. However, despite only a small proportion living in the same household, most (79%) regularly discussed their condition and its treatment with affected relatives. This raises the possibility that in an open-label, parallel group, randomized water intake trial, enrolling multiple members of the same family may lead to contamination of the control arm. Thus, careful consideration is required when selecting the randomization method for a water trial.

The strengths of our findings should be considered against its limitations. The questionnaire was accessible online via the Internet. The proportion of responders was low, and those who responded were predominantly British, Caucasian and female. Our results may therefore not be representative of male ADPKD patients or those from other ethnic backgrounds, and should be interpreted with caution. The low response rate (3.7%) is attributable to several factors: (i) invites were sent only once, given that patients on the mailing list receive frequent requests for surveys, (ii) we offered no incentive for participation and (iii) we restricted participation to online platforms. Nevertheless, the response rate was within the range reported from other online surveys [14].

Since high water intake should suppress vasopressin production, it may also slow the progression of ADPKD. Data from animal studies are consistent with this hypothesis, showing reduced cyst growth and slower decline in kidney function with increased hydration in a variety of rodent models [11, 15]. A number of small studies have looked at the impact of high water intake on PKD in humans. Higashihara *et al.* [12] allocated participants according to their preference to high (*n* = 18) and free (*n* = 16) water intake. Although not statistically significant, in the high intake group after 1 year the percentage change in TKV increased (3.8–9.1% per year) and the estimated glomerular filtration rate slope worsened (−0.3 to −7.1 mL/min/1.73 m^2^). In a pilot trial of 34 ADPKD patients [16], Amro *et al.* demonstrated that randomization to low osmolar diet and high fluid intake reduced plasma copeptin concentrations (a surrogate marker for vasopressin) and urine osmolality.

In unselected chronic kidney disease patients, a high water intake trial is ongoing and has achieved sufficient adherence to a high water intake prescription to demonstrate separation between trial arms [17]. In ADPKD, small studies suggest that water intake can be sufficiently increased to suppress vasopressin, rendering a large trial assessing renal outcomes feasible [12, 16, 18]. In a randomized pilot trial (PREVENT-ADPKD, ACTRN12614001216606), 180 ADPKD patients will be assigned to standard care or a high water intake prescription. The trial will assess feasibility endpoint and change in TKV. In a randomized feasibility trial (DRINK, NCT02933268), 50 ADPKD patients will be assigned to high versus *ad lib* water intake to assess feasibility, self-monitoring and treatment adherence. Together, these trials will inform the design of a definitive water intake trial in ADPKD. The SIPs survey data presented here provides preliminary data on the potential feasibility and acceptability of a high water intake trial in patients with ADPKD.

High water intake is an attractive intervention for ADPKD. It is readily available, generally safe and required to sustain life. It is likely to be suitable in patients excluded from or intolerant of pharmacological therapies. However, given the effect of renal impairment on water handling and conflicting data from existing literature, well-designed trials of high water intake in ADPKD are urgently needed. The SIPs survey results are an important first step towards achieving this goal.

## Supplementary data

Supplementary data are available online at http://ckj.oxfordjournals.org.

## Supplementary Material

Supplementary Data
