# Supplementary material for: Patient Survey of current water Intake practices in autosomal dominant Polycystic kidney disease: the SIPs survey
Source: Clin Kidney J. 2017 Feb 17;10(3):305–9. doi: 10.1093/ckj/sfw153 (PMC5466085; doi:10.1093/ckj/sfw153)
Supplement: Supplementary Data [file sfw153_Supp.pdf]

This free survey is powered by QuestionPro Create a Survey

Survey: Water Study

PKD Water Study

People with Autosomal Dominant Polycystic Kidney Disease (ADPKD) - who haven't progressed to kidney failure - are often asked to drink plenty of water throughout the day, in the hope that this would slow worsening of kidney function over time.

However, the effect of drinking large amounts of water on kidney function is unknown.

We are considering a trial of 'high' versus 'normal' water intake to determine whether high intake is beneficial or harmful.

Your views would be extremely valuable in ensuring that any such study is acceptable to most patients, maximally informative, and not unnecessarily burdensome.

This survey should take no more than 10 minutes and is totally anonymous.

Please note that this trial will be conducted on people at earlier stages of ADPKD. So if you already have had a transplant or on dialysis, you cannot take part in this survey. Sorry!

Are you male or female?

- ○ Male
- ○ Female

How old are you?

- ○ 15-19
- ○ 20-24
- ○ 25-29
- ○ 30-34
- ○ 35-39
- ○ 40-44
- ○ 45-49
- ○ 50-54
- ○ 55-59
- ○ 60-64
- ○ 65 or older
- ○ Rather not say

Which area of the UK do you live in?

- ○ East Midlands (Derbyshire, Nottinghamshire, Leicestershire, Northamptonshire, Lincolnshire, Rutland)
- ○ East of England (Norfolk, Suffolk, Cambridgeshire, Bedfordshire, Hertfordshire, Essex)
- ○ Greater London
- ○ North East England (Northumberland, County Durham, Tyne and Wear)
- ○ North West England (Cumbria, Lancashire, Cheshire, Merseyside, Greater Manchester)

- ○ Northern Ireland
- ○ Scotland
- ○ South Central England (Oxfordshire, Buckinghamshire, Hampshire, Isle of Wight, Berkshire)
- ○ South East Coast (Kent, East Sussex, West Sussex, Surrey)
- ○ South West England (Cornwall, Devon, Somerset, Dorset, Wiltshire, Gloucestershire)
- ○ Wales
- ○ West Midlands (Staffordshire, Shropshire, Herefordshire, Worcestershire, Warwickshire, Birmingham)
- ○ Yorkshire and the Humber (North Yorkshire, East Yorkshire, South Yorkshire, West Yorkshire)

Which of these ethnic groups would you say you belong to?

- ○ White (British, Irish, Traveller, or any other White background)
- ○ Mixed/Multiple Ethnic groups (White and Black Caribbean, White and Black African, White and Asian, any other Mixed background)
- ○ Asian or Asian British (Indian, Pakistani, Bangladeshi, Chinese, any other Asian background)
- ○ Black or Black British (Caribbean, African, or any other Black background)
- ○ Prefer not to say
- ○ Other ethnic group ______

What is your employment status?

- ○ Employed
- ○ Self-employed
- ○ Out of work
- ○ Homemaker
- ○ Student
- ○ Military
- ○ Retired
- ○ Other ______

If you are employed or self-employed, what is your occupation?

______

At what age were you diagnosed with ADPKD?

- ○ Under 14
- ○ 15-19
- ○ 20-24
- ○ 25-29
- ○ 30-34
- ○ 35-39
- ○ 40-44

- 45-49
- 50-54
- 55-59
- 60-64
- 65 or older
- Can’t recall

How often do you see a specialist in the hospital for the management of ADPKD?

Do you have other family members affected?

- Yes
- No
- Don't know

Do any family members live with you? The reason we ask this question is that family members may inadvertently influence your drinking habits, and this may affect the results of a study.

How often do you discuss your treatment/management with your affected family members (ranging from never to daily etc?)

ABOUT YOUR CURRENT WATER DRINKING HABITS

How much water - in litres - do you drink daily? You can include the water you drink in tea, coffee, soup and other beverages.

For reference, a typical cup of tea has about 250ml (quarter of a litre) of water.

- Less than 1 litre
- 1 - 1.5
- 1.5 - 2
- 2 - 2.5

- ○ 2.5 - 3
- ○ 3 - 3.5
- ○ 3.5 - 4
- ○ More than 4 litres

Do you actively make an effort to drink throughout the day even when you are not thirsty?

- ○ Yes
- ○ No

Do you get up at night to pass urine?

- ○ Always
- ○ Frequently
- ○ Occasionally
- ○ Never

To what extent do you believe drinking water throughout the day can slow down the progression of PKD?

In the context of a research study which is likely to last at least 3 years:

	Yes	No	Don't know
Would you agree to drinking large amounts of water (more than 3 litres per day)?	○	○	○
Would you agree to drink guided by thirst (for most people this is less than 2 litres)?	○	○	○

RESEARCH STUDY IMPLICATIONS

A few final questions

	Yes	No	Don't know
Would you be willing to provide a urine sample on a regular basis (every 1 to 2 months)?	○	○	○
Would you be willing to perform an occasional (3-4 times per year) 24 hour urine collection? This would be done at a time and place (eg home) convenient to you.	○	○	○
Would you be willing to have			

regular blood tests (after 3 months, then 6 monthly)?	○	○	○
Would you be willing to test your own urine at home using a coloured test strip and colour chart, and record the result on a computer or smartphone application?	○	○	○
Would you take part in research projects aimed at slowing ADPKD progression?	○	○	○

Do you have any comments/suggestions on this survey and the questions?

Share This Survey: Online Survey Software Powered by QuestionPro
